# Detecting Memory and Structure in Human Navigation Patterns Using Markov Chain Models of Varying Order

**DOI:** 10.1371/journal.pone.0102070

**Published:** 2014-07-11

**Authors:** Philipp Singer, Denis Helic, Behnam Taraghi, Markus Strohmaier

**Affiliations:** 1 GESIS - Leibniz Institute for the Social Sciences, Cologne, Germany; 2 Technical University of Graz, Knowledge Technologies Institute, Graz, Austria; 3 Technical University of Graz, Institute for Information Systems and Computer Media, Graz, Austria; 4 University Koblenz-Landau, Institute for Web Science and Technologies, Koblenz, Germany; Indiana University Bloomington, United States of America

## Abstract

One of the most frequently used models for understanding human navigation on the Web is the Markov chain model, where Web pages are represented as states and hyperlinks as probabilities of navigating from one page to another. Predominantly, human navigation on the Web has been thought to satisfy the memoryless Markov property stating that the next page a user visits only depends on her current page and not on previously visited ones. This idea has found its way in numerous applications such as Google's PageRank algorithm and others. Recently, new studies suggested that human navigation may better be modeled using higher order Markov chain models, i.e., the next page depends on a longer history of past clicks. Yet, this finding is preliminary and does not account for the higher complexity of higher order Markov chain models which is why the memoryless model is still widely used. In this work we thoroughly present a diverse array of advanced inference methods for determining the appropriate Markov chain order. We highlight strengths and weaknesses of each method and apply them for investigating memory and structure of human navigation on the Web. Our experiments reveal that the complexity of higher order models grows faster than their utility, and thus we confirm that the memoryless model represents a quite practical model for human navigation on a page level. However, when we expand our analysis to a topical level, where we abstract away from specific page transitions to transitions between topics, we find that the memoryless assumption is violated and specific regularities can be observed. We report results from experiments with two types of navigational datasets (goal-oriented vs. free form) and observe interesting structural differences that make a strong argument for more contextual studies of human navigation in future work.

## Introduction

Navigation represents a fundamental activity for users on the Web. Modeling this activity, i.e., understanding how predictable human navigation is and whether regularities can be detected has been of interest to researchers for nearly two decades – an example of early work would be work by Catledge and Pitkow [1]. Another example would be [2], who focused on trying to understand preferred user navigation patterns in order to reveal users' interests or preferences. Not only has our community been interested in gaining deeper insights into human behavior during navigation, but also in understanding how models of human navigation can improve user interfaces or information network structures [3]. Further work has focused on understanding whether models of human navigation can help to predict user clicks in order to prefetch Web sites (e.g., [4]) or enhance a site's interface or structure (e.g., [5]). More recently, such models have also been deployed in the field of recommender systems (e.g., [6]).

However, models of human navigation can only be useful to the extent human navigation itself exhibits regularities that can be exploited. An early study on user navigation in the Web by Huberman, Pirolli, Pitkow and Lukose [7], for example, already identified interesting regularities in the distributions of user page visits on a Web site. More recently, Wang and Huberman [8] confirmed these observations and Song, Qu, Blumm and Barabási [9] argued that the regularities in human activities might be based on the inherent regularities of human behavior in general.

The most prominent model for describing human navigation on the Web is the Markov chain model (e.g., [10]), where Web pages are represented as states and hyperlinks as probabilities of navigating from one page to another. Predominantly, the Markov chain model has been memoryless in a wide range of works (e.g., Google's PageRank [11]) indicating that the next state only depends on the current state of a user's Web trail. Recently, a study [12] suggested that human navigation might be better modeled with memory – i.e., the next page depends on a longer history of past clicks. However, this finding is preliminary and does not account for the higher complexity of higher order Markov chain models which is why the memoryless model is still widely used.

### Research questions

**Figure 1:**
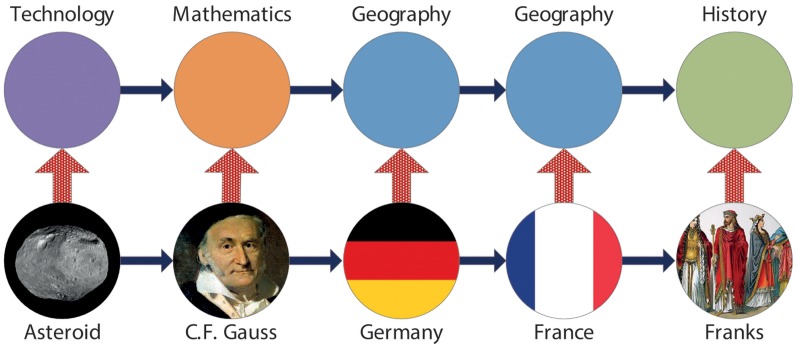
Example of a navigation sequence in the WikiGame dataset. Bottom row of nodes: A user navigates a series of Wikipedia articles, which can be represented as a sequence of Web pages. Top row of nodes: Each Wikipedia article can be mapped to a corresponding topic through Wikipedia's system of categories. This results in a sequence of topics.

In this paper, we are interested in shedding a deeper light on regularities in human navigation on the World Wide Web by studying memory and structure in human navigation patterns. We start by investigating memory of human navigational paths over Web sites by determining the order of corresponding Markov chains. We are specifically interested in detecting if the benefit of a larger memory (or higher order Markov chain) can compensate for the higher complexity of the model. In order to understand whether and to what extent human navigation exhibits memory on a topical level, we abstract away from specific page transitions and study memory effects on a topical level by representing click streams as sequences of topics (cf. Figure 1) – note that the terms “topic” and “category” should be seen as synonyms throughout this work. This enables us to (i) move up from the page to topical level and (ii) significantly reduce the complexity of higher order models and therefore (iii) gain deeper insights into memory and structure of human navigational patterns. Finally, we discuss our findings and demonstrate interesting differences between human navigation in free browsing vs. more goal-oriented settings.

### Methods and Materials

We study memory and structure in human navigation patterns on three similarly structured datasets: WikiGame (a navigation dataset with known navigation goals), Wikispeedia (another goal-oriented navigation dataset) and MSNBC (a free navigation dataset). For analyzing memory, we use Markov chains to model human behavior and analyze the appropriate Markov chain order – i.e., we investigate whether human navigation is memoryless or not. For model selection – i.e., the process of finding the most appropriate Markov chain order – we resort to a highly diverse array of methods stemming from distinct statistical schools: (i) likelihood [13], [14], (ii) Bayesian [15] and (iii) information-theoretic methods [14], [16]–[19]. We supplement these with a (iv) cross validation approach for a prediction task [18]. We thoroughly elaborate each method, put them into relation to each other and also highlight strengths and weaknesses of each. Such detailed derivation of model parameters and the model comparison is, for example, missing in previous work [12], which prevents us from drawing definite conclusions. We apply these methods to our human navigational data in order to get an exhaustive picture about memory in human navigation. Finally, we identify structural aspects by analyzing transition matrices produced by our Markov chain analyses.

### Contributions

The main contributions of this work are three-fold:

- First, we deploy four different, yet complementary, approaches for order selection of Markov chain models (likelihood, Bayesian, information-theoretic and cross validation methods) and elaborate their strengths and weaknesses. Hence, our work extends existing studies that model human navigation on the Web using Markov chain models [12]. By applying these methods on navigational Web data, our work presents – to the best of our knowledge – the most comprehensive and systematic evaluation of Markov model orders for human navigational sequences on the Web to date. Furthermore, we make our methods in the form of an open source framework available online (https://github.com/psinger/PathTools) to aid future work [20].
- Our empirical results confirm what we inferred from theory: It is difficult to make plausible statements about the appropriate Markov chain order having insufficient data but a vast amount of states, which is a common situation for Web page navigational paths. All evaluation approaches would favor a zero or first order because the number of parameters grows exponentially with the chain order and the available data is too sparse for proper parameter inferences. Thus, we show further evidence that the memoryless model seems to be a quite practical and legitimate model for human navigation on a page level.
- By abstracting away from the page level to a topical level, the results are different. By representing all datasets as navigational sequences of topics that describe underlying Web pages (cf. Figure 1), we find evidence that topical navigation of humans is not memoryless at all. On three rather different datasets of navigation – free navigation (MSNBC) and goal-oriented navigation (WikiGame and Wikispeedia) – we find mostly consistent memory regularities on a topical level: In all cases, Markov chain models of order two (respectively three) best explain the observed navigational sequences. We analyze the structure of such navigation, identify strategies and the most salient common sequences of human navigational patterns and provide visual depictions. Amongst other structural differences between goal-oriented and free form navigational patterns, users seem to stay in the same topic more frequently for our free form navigational dataset (MSNBC) compared to both of the goal oriented datasets (Wikigame and Wikispeedia). Our analysis thereby provides new insights into the memory and structure that users employ when navigating the Web that can e.g., be useful to improve recommendation algorithms, web site design or faceted browsing.

The paper is structured as follows: In the section entitled “Related Work” we review the state-of-the-art in this domain. Next, we present our methodology and experimental setup in the sections called “Methods” and “Materials”. We present and discuss our results in the section named “Results”. In the section called “Discussion we provide a final discussion and the section called “Conclusions” concludes our paper.

### Related Work

In the late 1990s, the analysis of user navigational behavior on the Web became an important and wide-spread research topic. Prominent examples are models by Huberman and Adamic [21] that determine how users choose new sites while navigating, or the work by Huberman, Pirolli, Pitkow and Lukose [7] who have shown that strong regularities in human navigation behavior exist and that, for example, the length of navigational paths on the Web is distributed as an inverse Gaussian distribution. These first models of human navigation on the Web set a standard modeling framework for future research - the majority of navigation models have been stochastic henceforth. Common stochastic models of human navigation are Markov chains. For example, the Random Surfer model in Google's PageRank algorithm can be seen as a special case of a Markov chain [11]. Some further examples of the application of Markov chains as models of Web navigation can be found in [10], [22]–[29].

In a Markov chain, Web pages are represented as states and links between the pages are modeled as probabilistic transitions between the states. The dynamics of a user's navigation session, in which she visits a number of pages by following the links between them, can thus be represented as a sequence of states. Specific configurations of model parameters – such as transition probabilities or model orders – have been used to reflect different assumptions about navigation behavior. One of the most influential assumptions in this field to date is the so-called Markovian property, which postulates that the next page that a user visits depends only on her current page, and not on any other page leading to the current one. This assumption is adopted in a number of prevalent models of human navigation in information networks, for example also in the Random Surfer model [11]. However, this property is neglecting the observations stated above that human navigation exhibits strong regularities which hints towards longer memory patterns in human navigation. We argue, that the more consistency human navigation in information networks displays the higher the appropriate Markov chain order should be.

#### The Markovian assumption might be wrong

The principle that human navigation might exhibit longer memory patterns than the first order Markov chain captures has been investigated in the past (see e.g., [3], [10] or [30] for a more general approach of looking at memory in network flows). However, higher order Markov chains have been often disputed for modeling human navigation because the gain of a higher order model did not compensate for the additional complexity introduced by the model [10]. Therefore, it was a common practice to focus on a first order model since it was a reasonable but extremely simple approximation of user navigation behavior (e.g., [25], [27], [28], [31]).

The discussion about the appropriate Markov chain order was just recently picked up again by Chierichetti, Kumar, Raghavan and Sarlos [12]. While the authors' results again show indicators that users on the World Wide Web are not Markovian, the study does not account for the higher complexity of such models and the possible lack of statistically significant gains of these models. Technically, the authors analyzed Markov chain models of different orders by measuring the likelihood of real navigational sequences given a particular model. In the next step, the authors compared the models by their likelihoods and found that the Markovian assumption does not hold for their given data and, thus, higher order Markov chain models seem to be more appropriate. As a result, the authors argue that users on the World Wide Web are not Markovian. However, their results come with certain limitations, such as the fact that choosing the model with the highest likelihood is biased towards models with more parameters. Because lower order models are always nested within higher order models and as higher order Markov chains have exponentially more parameters than lower order models (potential overfitting), they are always a better fit for the data [18]. Thus, higher order models are naturally favored by their improvements in likelihoods. A more comprehensive view on this issue shows that there exists a broad range of established model comparison techniques that also take into the account the complexity of a model in question [14]–[17], [19], [32], [33].

Moreover, the principle objects of interest in the majority of the past studies are transitions between Web pages. Only a few studies [27], [34], [35] investigate navigation as transitions between Web page features, such as the content or context of those Web pages.

## Methods

In the following, we briefly introduce Markov chains before discussing an expanded set of methods for order selection, including *likelihood*, *Bayesian*, *information-theoretic* and *cross validation* model selection techniques.

### Markov Chains

Formally, a discrete (time and space) finite Markov chain is a stochastic process which amounts to a sequence of random variables 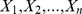. For a Markov chain of the first order, i.e., for a chain that satisfies the memoryless Markov property the following holds: 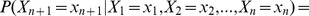


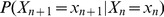
(1)


This classic first order Markov chain model is usually also called a *memoryless model* as we only use the current information for deriving the future and do not look into the past. For all our models we assume *time-homogeneity* – the probabilities do not change as a function of time. To simplify the notation we denote data as a sequence 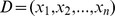 with states from a finite set 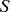. With this simplified notation we write the Markov property as: 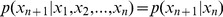
(2)


As we are also interested in higher order Markov chain models in this article – i.e., memory models – we now also define a Markov chain for an arbitrary order 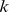 with 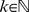 – or a chain with memory 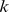. In a Markov chain of 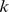-th order the probability of the next state depends on 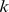 previous states. Formally, we write: 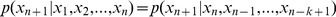
(3)


Markov chains of a higher order can be converted into Markov chains of order one in a straightforward manner – the set of states for a higher order Markov chain includes all sequences of length 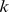 (resulting in a state set of size 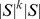). The transition probabilities are adjusted accordingly.

A Markov model is typically represented by a transition (stochastic) matrix 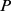 with elements 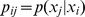. Since 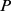 is a stochastic matrix it holds that for all 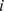: 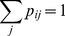
(4)


Please note, that for a Markov chain of order  the current state 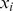 can be a compound state of length 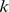 – it is a sequence of past 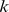 states. Throughout this paper we use this simpler notation, but one should keep in mind that 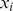 differs for distinct orders 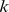.

For the sake of completeness, we also allow 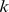 to be zero. In such a *zero order* Markov chain model the next state does not depend on any current or previous events, but simply can be seen as a *weighted random selection* – i.e., the probability of choosing a state is defined by how frequently it occurs in the navigational paths. This should serve as a baseline for our evaluations.

Next, we want to estimate the vector 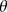 of parameters of a particular Markov chain that generated observed data 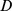 as well as determine the appropriate Markov chain order. For a Markov chain the model parameters are the elements 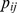 of the transition matrix 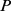, i.e., 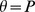.

### Model Selection

In this article our main goal is to determine the appropriate order of a Markov chain – i.e., the appropriate length of the memory. For doing so, we resort to well established statistical methods. As we want to provide a preferably complete array of methods for doing so, we present and apply methods from distinct statistical schools: (i) likelihood, (ii) Bayesian and (iii) information-theoretic methods. Note that no official classification of statistical schools is available; some may also argue that there are only the two competing schools of frequentists (which we do not explicitly discuss in this article) and Bayesians. The categorization used here is motivated by a short blog post (see http://labstats.net/articles/overview.html). We also supplement the methods coming from these three schools by providing a model selection technique usually known from machine learning: (iv) cross validation. We provide an overall ample view of methods and discuss advantages and limitations of each in the following sections.

### Likelihood Method

The term *likelihood* was coined and popularized by R. A. Fisher in the 1920's (see e.g, [13] for a historic recap of the developments). Likelihood can be seen as a central element of statistics and we will also see in the following sections that other methods also resort to the concept. The likelihood is a function of the parameters 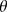 and it equals to the probability of observing the data given specific parameter values: 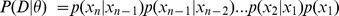


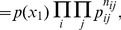
(5)


where 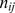 is the number of transition from state 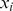 to state 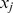 in 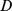.

Fisher also popularized the so-called *maximum likelihood estimate (MLE)* which has a very intuitive interpretation. This is the estimation of the parameters 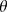 – i.e., transition probabilities – that most likely generated data 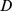. Concretely, the maximum likelihood estimate 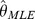 are the values of the parameters 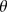 that maximize the likelihood function, i.e., 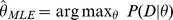 (a thorough introduction to MLE can be found in [36]).

The maximum likelihood estimation for Markov chains is an example of an optimization problem under constraints. Such optimization problems are typically solved by applying Lagrange multipliers. To simplify the calculus we will work with the log-likelihood function 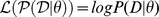. Because the 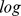 function is a monotonic function that preserves order, maximizing the log-likelihood is equivalent to maximizing the likelihood function. Thus, we have: 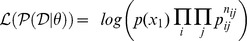


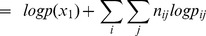
(6)


Our constraints capture the fact that each transition matrix row sums to 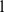: 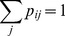
(7)


We have 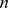 rows and therefore we need 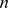 Lagrange multipliers 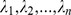. We can rewrite the constraints using Lagrange multipliers as: 
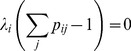
(8)


Now, the new objective function is: 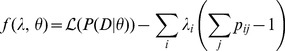
(9)


To maximize the objective function we set partial derivatives with respect to 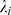 to 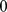, which gives back the original constraints.

Further, we set partial derivatives with respect to 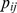 to 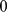 and solve the equation system for 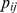. This gives: 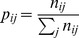
(10)


Thus, the maximum likelihood estimate for a specific 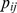 is the number of transitions from state 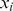 to state 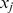 divided by the total number of transitions from state 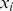 to any other state. For example, in a navigation scenario the maximum likelihood estimate for a transition from page 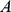 to page 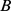 is the number of clicks on a link leading to page 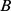 from page 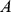 divided by the total number of clicks on page 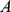.

**Figure 2:**
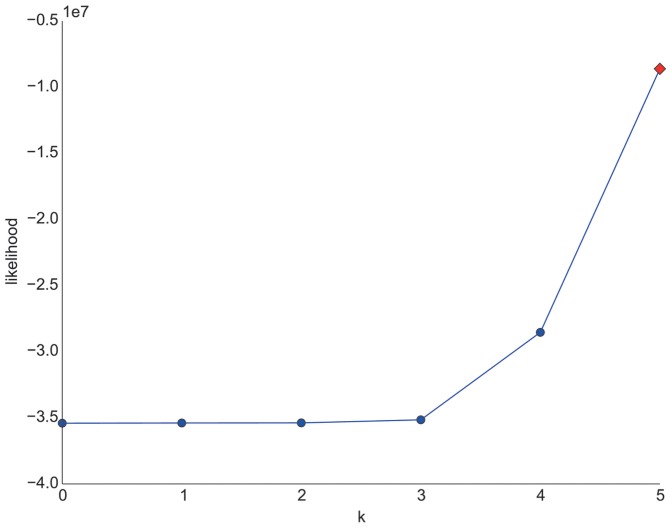
Log-likelihoods for random path dataset. Simple log-likelihoods of varying Markov chain orders would suggest higher orders as the higher the order the higher the corresponding log-likelihoods are. This suggests that looking at these log-likelihoods is not enough for finding the appropriate Markov chain order as methods are necessary that balance the goodness-of-fit against the number of model parameters.

Our concrete goal is to determine the appropriate order of a Markov chain. Using the log-likelihoods of the specific order models is not enough, as we will always get a better fit to our training data using higher order Markov chains. The reason for this is that lower order models are nested within higher order models. Also, the number of parameters increases exponentially with 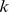 which may result in overfitting [18] since we can always produce better fits to the data with more model parameters. To demonstrate this behavior, we produced a random navigational dataset by randomly (uniformly) picking a next click state out of a list of arbitrary states. One of these states determines that a path is finished and a new one begins. With this process we could generate a random path corpus that is close to one main dataset of this work (Wikigame topic dataset explained in the section called “Materials”). Concretely, we as well chose 26 states and the same number of total clicks. Purely from our intuition, such a process should produce navigational patterns with an appropriate Markov chain order of zero or at maximum one. However, if we look at the log-likelihoods depicted in Figure 2 we can observe that the higher the order the higher the corresponding log likelihoods are.

This strongly suggests that – as previously explained – looking at the log-likelihoods is not enough for finding the appropriate Markov chain order. Hence, we first resort to a well-known statistical likelihood tool for comparing two models – the so-called *likelihood ratio test*.

This test is suited for comparing the fit of two composite hypothesis where one model – the so-called *null model* 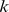 – is a special case of the *alternative model* 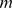. The test is based on the log likelihood ratio, which expresses how much more likely the data is with the alternative model than with the null model. We follow the notation provided by Tong [14] and denote the ratio as 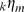: 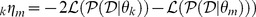
(11)


To address the overfitting problem we perform a significance test on this ratio. The significance test recognizes whether a better fit to data comes only from the increased number of parameters. The test calculates the p-value of the likelihood ratio distribution. Whenever the null model is nested within the alternative model the likelihood ratio approximately follows a 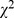 distribution with degrees of freedom specified by 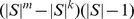. If the p-value is below a specific significance level we can reject the null hypothesis and prefer the alternative model [32] – note that this method also utilizes mechanisms usually known from the frequentist school; i.e., hypothesis testing.

Likelihood ratios and corresponding tests have been shown to be a very understandable approach of specifying evidence [37]. They also have the advantage of specifying a clear value (i.e., the likelihood ratio) with can give us intuitive meaning about the advantage of one model over the other. However, the likelihood-ratio test also has limitations like that it only works for nested models, which is fine for our approach but may be problematic for other use cases. It also requires us to use elements from frequentist approaches (i.e., the p-value) for deciding between two models which have been criticized in the past (e.g., [38]). Furthermore, we are only able to compare two models with each other at a time. This makes it difficult to choose one single model as the most likely one as we may end up with several statistical significant improvements. Also, as we increase the number of hypothesis in our test, we as well increase the probability that we find at least one significant result (Type 1 error). We could tackle this problem by e.g., applying the *Bonferroni correction* which we leave open for future work.

### Bayesian Method

Bayesian inference is a statistical method utilizing the Bayes' rule – Rev. Thomas Bayes started to talk about the Bayes theorem in 1764 – for updating prior believes with additional evidence derived from data. A general introduction to Bayesian inference can e.g., be found in [39]; in this article we focus on explaining the application for deriving the appropriate Markov chain order (see [15] for further details).

In Bayesian inference data and the model parameters are treated as random variables (cf. MLE where parameters are unknown constants). We start with a joint probability distribution of data 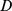 and parameters 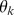 given a model 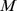; that is given a Markov chain of a specified order 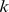. Thus, we are interested in 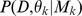.

The joint distribution 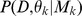 can be written as the product of the conditional probability of data 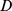 given the parameters 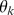 and the marginal distribution of the parameters, or we can write this joint distribution as the product of the conditional probability of the parameters given the data and the marginal distribution of the data.

Solving then for the posterior distribution of parameters given data and a model we obtain the famous Bayes rule: 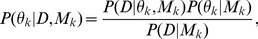
(12)


where 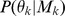 is the prior probability of model parameters, 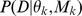 is the likelihood function; that is the probability of observing the data given the parameters, and 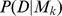 is the evidence (marginal likelihood). 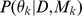 is the posterior probability of the parameters, which we obtain after we update the prior with the data.

For a more detailed and an in-depth technical analysis of Bayesian inference of Markov chains we point to an excellent discussion of the topic in [15].

#### Likelihood

As previously, we have: 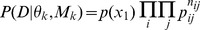
(13)


#### Prior

The prior reflects our (subjective or objective) belief about the parameters before we see the data. In Bayesian inference, conjugate priors are of special interest. Conjugate priors result in posterior distributions from the same distribution family. In our case, each row of the transition matrix follows a categorical distribution. The conjugate prior for categorical distribution is the Dirichlet distribution. Further information on applying Dirichlet conjugate prior and dealing with Dirichlet process can be found in [40]. The Dirichlet distribution is defined as 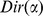: 
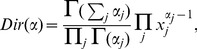
(14)


where 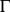 is the gamma function, 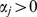 for each 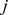 and 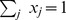 is a probability simplex. The probability outside of the simplex is 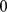.

The *hyperparameters* 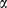 reflect our assumptions about the parameters 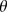 before we have observed the data. We can think about the hyperparameters as fake counts in the transition matrix of a Markov chain. A standard uninformative selection for hyperparameters is a uniform prior – for example, we set 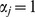 for each 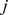.

Thus, for row 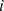 of the transition matrix we have the following prior: 
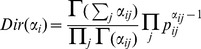
(15)


As before, it holds that: 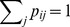
(16)


The prior for the complete transition matrix is the product of the Dirichlet distributions for each row: 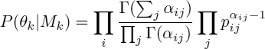
(17)


#### Evidence

To calculate the evidence we take a weighted average over all possible values of the parameters 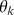. Thus, we need to integrate out the parameters 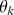. 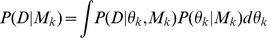
(18)

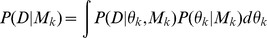


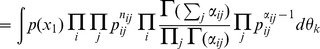


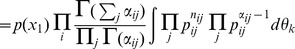


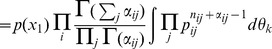



Please note, that: 
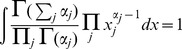


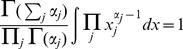


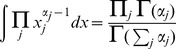



Thus, we have 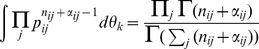
(19)


And thus, 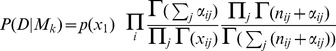
(20)


#### Posterior

For the posterior distribution over the parameters 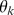 we obtain: 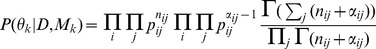


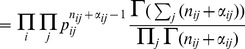



This equation is the product of the Dirichlet distributions for each row with parameters 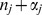:
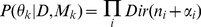
(21)


The posterior distribution is a combination of our prior belief and the data that we have observed. In fact, the expectation and the variance of the posterior distribution are: 
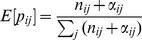
(22)

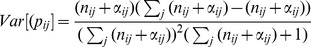
(23)


We can rewrite the expectation as: 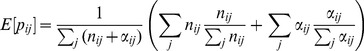
(24)


Setting 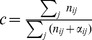, we can rewrite the expectation of the posterior distribution as: 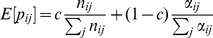
(25)


Thus, the posterior expectation is a *convex combination* of the MLE and the prior. When the number of the observation becomes large (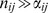) then 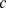 tends to 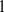, and the posterior expectation tends to the MLE.

By setting 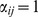 for each 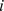 and 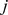 we effectively obtain Laplace's prior; that is we apply Laplace smoothing [18].

For model selection we adopt once more the Bayesian inference (again see [15] for a thorough discussion). We have a set 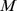 of models 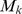 with varying order 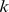 and are interested in deciding between several models (c.f. [41]). We are interested in the joint probability distribution 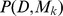 of data 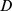 and a model 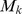. We can write the joint distribution as a product of a conditional probability (of data given a model, or of a model given the data) and a prior marginal distribution (of data or a model) and by solving for the posterior distribution of a model given the data we again obtain the Bayes rule: 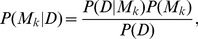
(26)


where 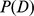 is the weighted average over all models 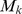: 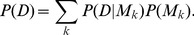
(27)


The likelihood of data 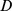 given a model 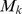 is the evidence 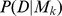 given by Equation 20, which is the weighted average over all possible model parameters 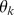 given the model 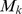.

Following Strelioff, Crutchfield and Hübler [15], we select two priors over the model set 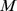 – a uniform prior and a prior with an exponential penalty for the higher order models [15]. The uniform prior assigns the identical probability for each model: 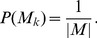
(28)


With the uniform prior we obtain the following expression for the posterior probability of a model 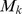 given the data: 
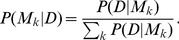
(29)


The prior with the exponential penalty can be defined as: 
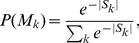
(30)


where 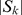 is the number of states of the model 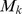 and can be calculated as: 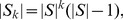
(31)


with 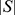 being the number of states of the model of order 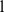.

After solving for the posterior distribution for the prior with the exponential penalty we obtain: 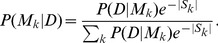
(32)


The calculations are best implemented with log-evidence and logarithms of the gamma function to avoid underflow since the numbers are extremely small. To implement the sum for the normalizing constant in the denominator we apply the so-called *log-sum-exp trick* [42]. First, we calculate the log-evidence: 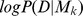 and then calculate the logarithm of the normalizing constant 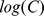: 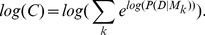
(33)


A direct calculation of 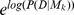 results in an underflow, and thus we pull the largest log-evidence 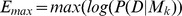 out of the sum: 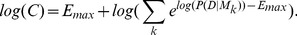
(34)


One downside of using Bayesian model selection is that it is frequently difficult to calculate Bayes factors. Concretely, it is often complicated to calculate the necessary integral analytically and one needs to resort to various alternatives in order to avoid this problem. Nowadays, several such methods exist: e.g., asymptotic approximation or sampling from the posterior (MCMC, Gibbs) [41]. Also, we need to specify prior distributions for the parameters of each model. As elaborated by Kass and Raftery [41], one approach is to use the BIC (see the next section entitled “Information-theoretic Methods”) which gives an appropriate approximation given one specific prior.

Compared to the likelihood ratio test (see section entitled Likelihood Method), the Bayesian model selection technique does not require the models to be nested. The main benefit of Bayesian model selection is that it includes a natural *Occam's razor* – i.e., a penalty for too much complexity – which helps us to avoid overfitting [41], [43]–[45]. The Occam's razor is a principle that advises to prefer simpler theories over more complex ones. Based on this definition there is no need to include extra complexity control as we e.g., additionally did for our exponential penalty. We see this though as a nice further control mechanism for cautiously penalizing model complexity and for validating the natural Occam's razor.

### Information-theoretic Methods

Information-theoretic methods are based on concepts and ideas derived from information theory with a specific focus on *entropy*. In the following we will provide a description of the two probably most well-known methods; i.e., AIC and BIC. A thorough overview of information-theoretic methods can e.g., be found in various work by K. P. Burnham [46], [47].

#### Akaike information criterion (AIC)

Akaike [16] introduced in 1973 a one dimensional statistic for determining the optimal model from a class of competing models. The criterion is based on Kullback-Leibler divergence [48] and the asymptotic properties of the likelihood ratio statistics described in the section entitled “Likelihood Method”. The approach is based on minimization of AIC (minimum AIC estimate – MAICE) amongst several competing models [33] and has been first used for Markov chains by Tong [14]. Hence, we define the AIC based on the choice of a loss function proposed by Tong [14]: 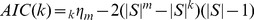
(35)


The test represents an asymptotic version of the likelihood ratio test defined in Equation 11 for composite hypothesis. The idea is to choose 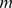 reasonably high and test lower order models until an optimal order is found. MAICE chooses the order 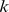 which exhibits the minimum AIC score and tries to balance between overfitting and underfitting [33].

#### Bayesian Information Criterion (BIC)

In 1978 Schwarz [19] introduced this criterion which can be seen as an approximation of the Bayes factor for Bayesian model selection (see the previous section entitled “Bayesian Method”). It is similar to the AIC introduced above with the difference that it penalizes higher order models even more by adding an additional penalization for the number of observations [17]: 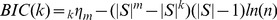
(36)


Again we choose 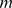 reasonably high and test lower order models against it. The penalty function is the degree of freedom multiplied with the natural logarithm of the number of observations 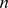. This function converges to infinity at a still slow enough rate and hence, grants a consistent estimator of the Markov chain order [17].

Frequently, both AIC and BIC suggest the same model. However, there are certain cases, where they might slightly disagree. In model selection literature there is a still ongoing debate of whether one should prefer AIC or BIC over each other – e.g., see [49] for a critique of the BIC for model selection. However, as pointed out by Burnham and Anderson [47], each has its strength and weaknesses in distinct domains. The authors emphasize that both can be seen as either frequentist or Bayesian procedures. In case of inequality, Katz [17] suggests to investigate the patterns further by simulating observations and investigate distinct sample sizes. In this paper we instead apply additional model comparison techniques to further analyze the data.

The performance of AIC and BIC has also been investigated in the terms of determining the appropriate Markov chain order which is the main goal of this article. R. W. Katz [17] pointed out that by using AIC there is the possibility of overestimating the true order independent of how large the data is. Hence, he points out that AIC is an inconsistent method. Contrary, he emphasizes that BIC is a consistent estimator – i.e., if there is a true underlying model BIC will select it with enough data. Alas, it does not perform well for small sample sizes (see also [50]). Nonetheless, AIC is the most used estimator for determining the appropriate order, maybe due to higher efficiency for smaller data samples, as elaborated by Baigorri, Gonçalves and Resende [51].

While both AIC and BIC seem at first to be very similar to the likelihood ratio test (see section entitled “Likelihood Method) there are some elementary differences. First and foremost, they can also be applied for non-nested models [46]. Moreover, they do not need to resort to hypothesis testing. BIC is also closely related to Bayesian model selection techniques; specifically to the Bayes factor (see section called “Bayesian Method”). Kass and Raftery [41] emphasize the advantages of BIC over the Bayes factor by pointing out that it can be applied even when the priors are hard to set. Also, it can be a rough approximation to the logarithm of the Bayes factor if the number of observations is large. BIC is also declared as being well suited for scientific reporting.

Finally, we want to point out that one could also see AIC as being best for prediction, while BIC might be better for explanation. Also, as pointed out by M. Stone [52], AIC is asymptotically equivalent to cross validation (see the section entitled “Cross Validation Method”) if both use maximum likelihood estimation.

### Cross Validation Method

Another – quite natural – way of determining the appropriate order of a Markov chain is cross-validation [12], [18]. The basic idea is to estimate the parameters on a training set and validate the results on an independent testing set. In order to reduce variance we perform a stratified 10-fold cross-validation. In difference to a classic machine learning scenario, we refer to stratified as a way of keeping approximately the equal amount of observations in each fold. Thus, we keep approximately 10% of all clicks in a single fold.

With this method we focus on prediction of the next user click. Markov chains have been already used to prefetch the next page that the user most probably will visit on the next click. In the simplest scenario, this prefetched page is the page with the highest transition probability from the current page. To measure the prediction accuracy we measure the average rank of the actual page in sorted probabilities from the transition matrix. Thus, we determine the rank of the next page 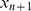 in the sorted list of transition probabilities (expectations of the Bayesian posterior) of the current page 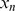 (see the section named “Markov Chains”). We then average the rank over all observations in the testing set. Hence, we can formally define the average rank 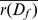 of a fold 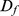 for some arbitrary model 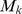 the following way: 
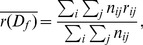
(37)


where 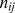 is the number of transition from state 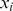 to state 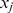 in 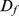 and 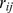 denotes the rank of 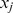 in the 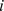-th row of the transition matrix.

For ranking the states in a row of the matrix, we resort to *modified competition ranking*. This means that if there is a tie between two or more values, we assign the maximum rank of all ties to each corresponding one; i.e., we leave the gaps before a set of ties (e.g., “14445” ranking). By doing so, we assign the worst possible ranks to ties. One important implication of this methodology is that we include a natural penalty (a natural Occam's razor) for higher order Markov chains. The reason for this is that the transition matrices generally become sparser the higher the order. Hence, we come up with many more ties and the chance is higher that we assign higher ranks for observed transitions in the testing data. The most extreme case happens when we do not have any information available for observations in the testing set (which frequently happens for higher orders); then we assign the maximum rank (i.e., the number of states) to all states. We finally average the ranks over all folds for a given order and suggest the model with the lowest average rank. In order to confirm our findings we also applied an additional way of determining the accuracy which is motivated by a typical evaluation technique known from link predictors [53]. Concretely, it counts how frequently the true next click is present in the TopK (k = 5) states determined by the probabilities of the transition matrix. In case of ties in the TopK elements we randomly draw from the ties. By applying this method to our data we can mirror the evaluation results obtained by using the described and used ranking technique. Note that we do not explicitly report the additional results of this evaluation method throughout the paper.

This method requires priors (i.e., fake counts; see the section named “Bayesian Method”) – otherwise prediction of unseen states is not possible. It also resorts to the maximum likelihood estimate for calculating the parameters of the models as described in the section entitled “Likelihood Method”. Also, as shown in the previous section called “Information-theoretic Methods” cross validation has asymptotic equivalence to AIC.

One disadvantage of cross validation methods usually is that the results are dependent on how one splits the data. However, by using our stratified k-fold cross validation approach, we counteract this problem as it matters less of how the data is divided. Yet, by doing so we need to rerun the complete evaluation k times, which leads to high computational expenses compared to the other model selection techniques described earlier and we have to manually decide of which k to use. One main advantage of this method is that eventually each observation is used for both training and testing.

## Materials

In this paper, we perform experiments on three datasets. While the first two datasets (WikiGame and Wikispeedia) are representatives of goal-oriented navigation scenarios (where the target node for each navigation sequence is known beforehand), the third dataset (MSNBC) is representative of free navigation on the Web (where we have no knowledge about the targets of navigation).

### Wikigame dataset

This dataset is based on the online game *TheWikiGame* (http://thewikigame.com/). The game platform offers a multiplayer game, where users navigate from a randomly selected Wikipedia page (the start page) to another randomly selected Wikipedia page (the target page). All pairs of start and target pages are connected through Wikipedia's underlying network. The users are only allowed to click on Wikipedia links or on the browser back button to reach the target page, but they are not allowed to use search functionality.

In this study, we only considered click paths of length two or more going through the main article namespace in Wikipedia. Table 1 shows some main characteristics of our Wikigame dataset.

**Table 1:** Dataset statistics.

	Wikigame	Wikispeedia	MSNBC
#Page Ids	360,417	n/a	n/a
#Topics	25	15	17
#Paths	1,799,015	43,772	624,383
#Visited nodes	10,758,242	259,019	4,333,359

**Figure 3:**
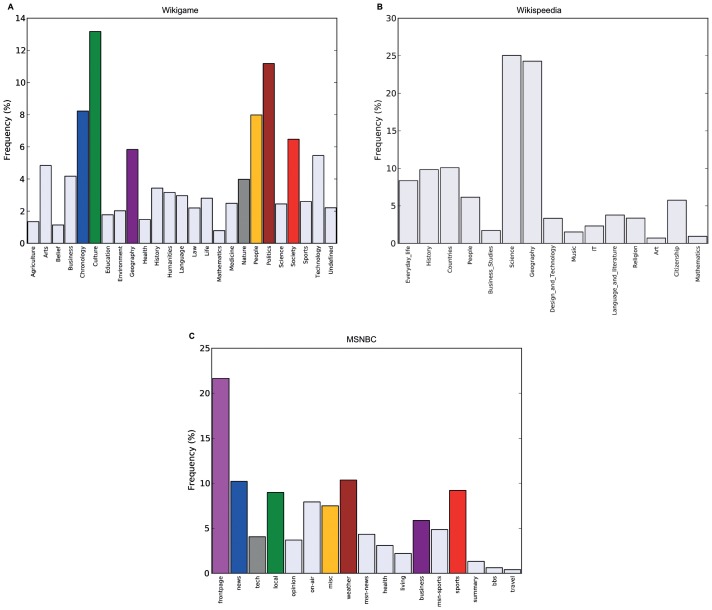
Topic frequencies. Frequency of categories (in percent) of all paths in (A) the Wikigame topic dataset (B) the Wikispeedia dataset and (C) the MSNBC dataset. The colors indicate the categories we will investigate in detail later and are representative for a single dataset – this means that the same color in the datasets does not represent the same topic. The Wikigame topic dataset consists of more distinct categories than the Wikispeedia and MSNBC dataset. Furthermore, the most frequently occuring topic in the Wikigame topic dataset is Culture with around 13%. The Wikispeedia dataset is dominated by the two categories the most Science and Geography each making up for almost 25% of all clicks. Finally, the most frequent topic in the MSNBC dataset is the frontpage with a frequency of around 22%.

As motivated in Section “Introduction”, we will represent the navigational paths through Wikipedia twofold: (a) each node in a path is represented by the corresponding Wikipedia page ID – we refer to this as the *Wikigame page* dataset – and (b) each node in a path is represented by a corresponding Wikipedia category (representing a specific topic) – we call this the *Wikigame topic* dataset. For the latter dataset we determine a corresponding top level Wikipedia category (http://en.wikipedia.org/wiki/Category:Main_topic_classifications) in the following way. The majority of Wikipedia pages belongs to one or more Wikipedia categories. For each of these categories we find a shortest path to the top level categories and select a top level category with the shortest distance. In the case of a tie we pick a top level category uniformly at random. Finally, we replace all appearances of that page with the chosen top level category. Thus, in this new dataset we replaced each navigational step over a page with an appropriate Wikipedia category (topic) and the dataset contains paths of topics which users visited during navigation (see Figure 1). Figure 3 illustrates the distinct topics and their corresponding occurrence frequency (A).

### Wikispeedia dataset

This dataset is based on a similar online game as the Wikigame dataset called *Wikispeedia* (http://www.cs.mcgill.ca/~rwest/wikispeedia/). Again, the players are presented with two randomly chosen Wikipedia pages and they are as well connected via the underlying link structure of Wikipedia. Furthermore, users can also select their own start and target page instead of getting randomly chosen ones. Contrary to the Wikigame, this game is no multiplayer game and you do not have a time limit. Again, we only look at navigational paths with at least two nodes in the path. The main difference to the Wikigame dataset is that Wikispeedia is played on a limited version of Wikipedia (Wikipedia for schools http://schools-wikipedia.org/) with around 4,600 articles. Some main characteristics are presented in Table 1. Conducted research and further explanations of the dataset can be found in [35], [54]–[56].

As we want to look at transitions between topics we determine a corresponding top level category (topic) for each page in the dataset. We do this in similar fashion as for our Wikigame dataset, but the Wikipedia version used for Wikispeedia has distinct top level categories compared to the full Wikipedia. Figure 3 illustrates the distinct categories and their corresponding occurrence frequency (B).

### MSNBC dataset

This dataset (http://kdd.ics.uci.edu/databases/msnbc/msnbc.html) consists of Web navigational paths from MSNBC (http://msnbc.com) for a complete day. Each single path is a sequence of page categories visited by a user within a time frame of 24 hours. The categories are available through the structure of the site and include categories such as *news*, *tech*, *weather*, *health*, *sports*, etc. In this dataset we also eliminate all paths with just a single click. Table 1 shows the basic statistics for this dataset and in Figure 3 the frequency of all categories of this dataset are depicted (C).

### Data preparation

Each dataset 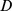 consists of a set of paths 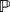. A single path contains a single game in the Wikigame and Wikispeedia dataset or a single navigation session in the MSNBC dataset. A path 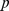 is defined as a 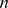-tuple 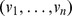 with 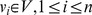 and 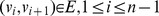 where 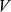 is the set of all nodes in 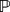 and 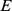 is the set of all observed transitions in 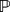. We also define the length of a path 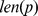 as the length of the corresponding tuple 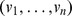. Additionally, we want to define 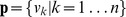 as the set of nodes in a path 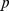. Note that 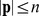. The finite state set 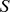 needed for Markov chain modeling is originally the set of vertices 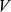 in a set of paths 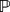 given a specific dataset 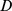. To prepare the paths for estimation of parameters of a Markov chain of order 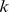, we separate single paths by prepending a sequence of 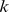 generic *RESET* states to each path, and also by appending one *RESET* state at the end of each path. This enables us to connect independent paths and – through the addition of the *RESET* state – to forget the history between different paths. Hence, we end up with an ergodic Markov chain (see [12]). With this artificial *RESET* state, the final number of states is 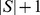.

## Results

In this section we present the results obtained from analyzing human navigation patterns based on our datasets at hand introduced in Section “Materials”. We begin by presenting the results of our investigations of memory – i.e., appropriate Markov chain order using the Markov chain methods thoroughly explained in the section called “Methods” – of user navigation patterns in the section entitled “Memory”. Based on these calculations and observations we dig deeper into the structure of human navigation and try to find consistent patterns – i.e., specific sequences of navigated states – in the section named “Structure”.

### Memory

We start by analyzing human navigation over Wikipedia pages on the Wikigame page dataset. Afterwards, we will focus on our topic datasets for getting insights on a topical level.

### Page navigation

#### Wikigame page dataset

**Figure 4:**
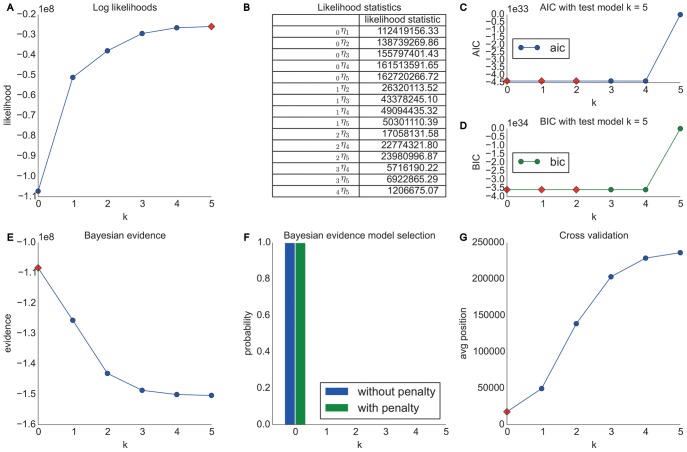
Model selection results for the Wikigame page dataset. The top row shows results obtained using likelihood and information theoretic results: (A) likelihoods, (B) likelihood ratio statistics (* statistically significant at the 1% level; ** statistically significant at the 0.1% level) as well as AIC (C) and BIC (D) statistics. The bottom row illustrates results obtained from Bayesian Inference: (E) evidence and (F) Bayesian model selection. Finally, the figure presents the results from (G) cross validation. The overall results suggest a zero order Markov chain model.

The initial Markov chain model selection results (see Figure 4) obtained from experiments on the Wikigame page dataset confirm our theoretical considerations. We observe that the likelihoods are rising with higher Markov chain orders (confirming what [12] found) which intuitively would indicate a better fit to the data using higher order models. However, the likelihood grows per definition with increasing order and number of model parameters and therefore, the likelihood based methods for model selection fail to penalize the increasing model complexity (c.f. Section “Likelihood Method”). All other applied methods take the model complexity into account.

First, we can imply already from the likelihood statistics (B) that there might be no improvement over the most basic zero order Markov chain model as we can not find any statistically significant improvements of higher orders. Both AIC (C) and BIC (D) results confirm these observations and also agree with each other. Even though we can see equally low values for a zero, first and second order Markov chain, we would most likely prefer the most simple model in such a case – further following the ideas of the Occam's razor.

In order to extend these primary observations we used a uniform Laplace prior and Bayesian inference and henceforth, we obtain the results illustrated in the first two figures of the bottom row in Figure 4. The Bayesian inference results again suggest a zero order Markov chain model as the most appropriate as indicated by the highest evidence (E) and the highest probability obtained using Bayesian model selection with and without a further exponential penalty for the number of parameters (F).

The observations and preference of using a zero order model are finally confirmed by the results obtained from using 10-fold cross-validation and a prediction task (G). We can see that the average position is the lowest for a zero order model approving our observations made above.

#### Summary

Our analysis of the Wikigame page dataset thereby reveals a clear trend towards a zero order Markov chain model. This is imminent when looking at all distinct model selection techniques introduced and applied in this article, as they all agree on the choice of weighted random selection as the statistically significant most approvable model. This is a strong approval of our initial hypothesis stating it is highly difficult to make plausible statements about the appropriate Markov chain order having insufficient data but a vast amount of states. The higher performance of higher order chains can not compensate the necessary additional complexity in terms of statistically significant improvements. However, this may be purely an effect of the data sparsity in our investigation (i.e., the limited number of observations compared to the huge amount of distinct states). One can argue that real human navigation always can be better modeled by at least an order of one, because – as soon as we have enough data – links play a vital role in human navigation as humans by definition follow links when they navigate – except for teleportation which we do not model in this work. Consequently, we believe that the memoryless Markov chain model is a plausible model for human navigation on a page level. Yet, further detailed studies are necessary to confirm this.

At the same time, one could argue that memory is best studied on a topical level, where pages are represented by topics. Consequently, we focus on studying transitions between topics next, which yields a reduced state space that allows analysis of the memory and structure of human navigation patterns on a topical level.

### Topics navigation

#### Wikigame topic dataset

**Figure 5:**
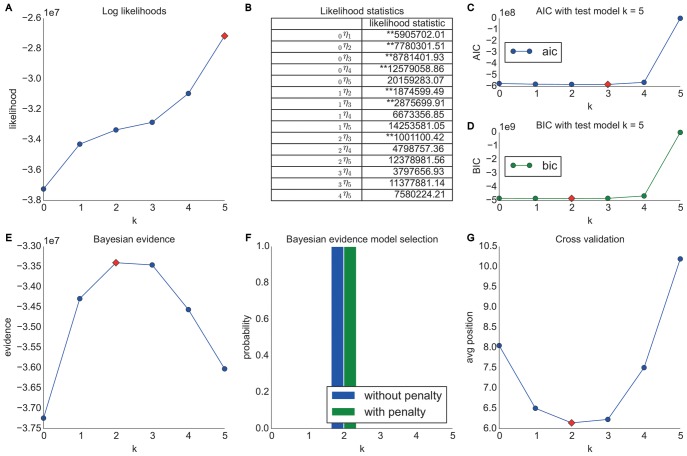
Model selection results for the Wikigame topic dataset. The top row shows results obtained using likelihood and information theoretic results: (A) likelihoods, (B) likelihood ratio statistics (* statistically significant at the 1% level; ** statistically significant at the 0.1% level) as well as AIC (C) and BIC (D) statistics. The bottom row illustrates results obtained from Bayesian Inference: (E) shows evidence and (F) Bayesian model selection. (G) presents the results from cross validation. The overall results suggest that higher order chains seem to be more appropriate for our navigation paths consisting of topics. In detail, we find that a second order Markov chain model for our Wikigame topic dataset best explains the data.

Performing our analyses by representing Wikipedia pages by their topical categories shows a much clearer and more interesting picture as one can see in Figure 5. Similar to above we can see (A) that the log likelihoods are rising with higher orders. However, in contrast to the Wikigame page dataset, we can now see (B) that several higher order Markov chain models are significantly better than lower orders. In detail, we can see that the appropriate Markov chain order is at least of order one and we can also observe a trend towards an order of two or three. Nevertheless, as pointed out in the section entitled “Likelihood Method”, it is hard to concretely suggest one specific Markov chain order from these pairwise comparisons which is why we resort to this extended repertoire of model selection techniques described next.

The AIC (C) and BIC (D) statistics show further indicators – even though they are disagreeing – that the appropriate model is of higher order. Concretely, the suggest an order of three or two respectively by exhibiting the lowest values at these points. Not surprisingly, AIC suggests a higher order compared to BIC as the latter model selection method additionally penalized higher orders by the number of observations as stated in the section called “Information-theoretic Methods”.

The Bayesian inference investigations (E, F) exhibit a clear trend towards a Markov chain of order two. The results in (F) nicely illustrate the inherent Occam's razor of the Bayesian model selection method as both priors – (a) no penalty and (b) exponential penalty for higher orders – suggest the same order (both priors agree throughout all our investigations in this article). Finally, the cross validation results (G) confirm that a second order Markov chain produces the best results, while a third order model is nearly as good.

#### Summary

Overall, we can see that representing Wikigame paths as navigational sequences of corresponding topics leads to more interesting results: Higher order Markov chains exhibit statistically significant improvements, thereby suggesting that memory effects are at play. Overall, we can suggest that a second order Markov chain model seems to be the most appropriate for modeling the corresponding data as it gets suggested by all methods except for AIC which is known for slightly overestimating the order. This means, that humans remember their topical browsing patterns – in other words, the next click in navigational trails is dependent on the previous two clicks on a topical level.

#### Wikispeedia dataset

**Figure 6:**
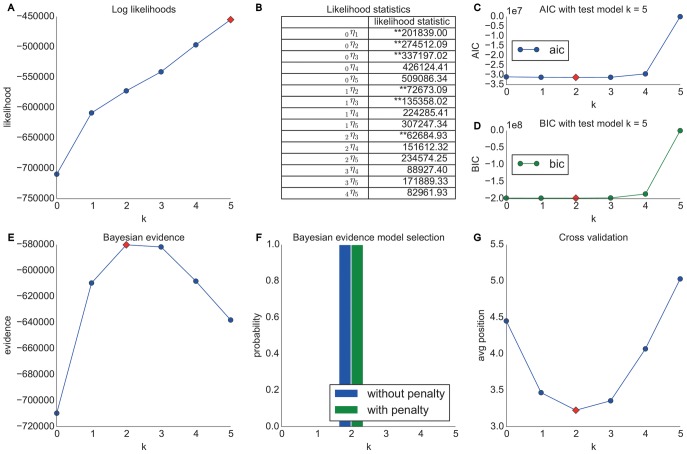
Model selection results for the Wikispeedia dataset. The top row shows results obtained using likelihood and information theoretic results: (A) likelihoods, (B) likelihood ratio statistics (* statistically significant at the 1% level; ** statistically significant at the 0.1% level) as well as AIC (C) and BIC (D) statistics. The bottom row illustrates results obtained from Bayesian Inference: (E) shows evidence and (F) Bayesian model selection. (G) presents the results from cross validation. The overall results suggest that higher order chains seem to be more appropriate for our navigation paths consisting of topics. Concretely, we find that a second order Markov chain model for our Wikispeedia topic dataset best explains the data.

This section presents the results obtained from the Wikispeedia dataset introduced in the section entitled “Materials”. Similar to the Wikigame topic dataset we look at navigational paths over topical categories in Wikipedia and present the results in Figure 6. Again we can observe that the likelihood statistics suggest higher order Markov chains to be appropriate (B). Yet, further analyses are necessary for a clear choice of the appropriate order. The AIC (C) and BIC (D) statistics agree to prefer a second order model; however, we need to note that all orders from zero to four have similarly low values. The Bayesian inference investigations (E, F) show a much clearer trend towards a second order model. The prediction results (G) agree on these observations by also showing the best results for a second order model. This time we can also observe a clear consilience between the cross validation and AIC results which are – as described in the section called “Information-theoretic Methods” – asymptotically equivalent.

#### Summary

This dataset is similar to the Wikigame topic dataset and the results are comparable to the previous results on the first goal-oriented dataset (Wikigame topic). Hence, even though the game is played on a much smaller set of Wikipedia articles and also the dataset consists of distinct categories, we can see the exact same behavior which strongly indicates that human navigation is not memoryless on a topical level and can be best modeled by a second order Markov chain model. This strongly suggests that humans follow common topical strategies while navigating in a goal-oriented scenario.

#### MSNBC dataset

**Figure 7:**
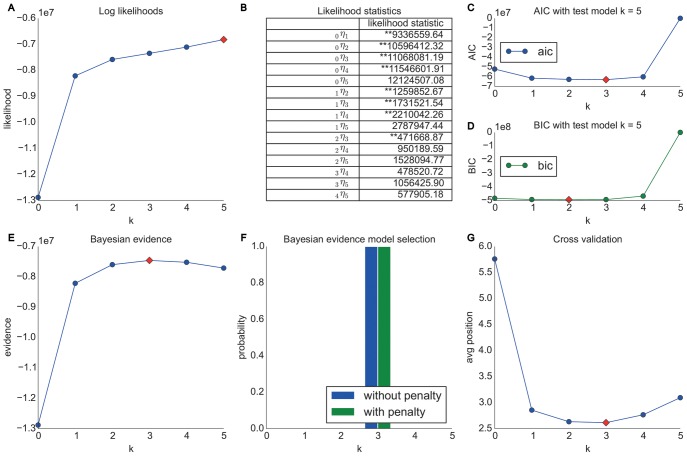
Model selection results for the MSNBC dataset. The top row shows results obtained using likelihood and information theoretic results: (A) likelihoods, (B) likelihood ratio statistics (* statistically significant at the 1% level; ** statistically significant at the 0.1% level) as well as AIC (C) and BIC (D) statistics. The bottom row illustrates results obtained from Bayesian Inference: (E) shows evidence and (F) Bayesian model selection. (G) presents the results from cross validation. The overall results suggest that higher order chains seem to be more appropriate for our navigation paths consisting of topics. Specifically, the results suggest a third order Markov chain model.

In this section we present the results obtained from the MSNBC dataset introduced in the section called “Materials”. Again we look at navigational paths over topical categories and henceforth, we only look at categorical information of nodes and present the results in Figure 7.

Similar to the experiments conducted for the Wikigame and Wikispeedia topic datasets we can again see, based on the likelihood ratio statistics (B), that a higher order Markov chain seems to be appropriate. The AIC (C) and BIC (D) statistics suggest an order of three and two respectively. To further investigate the behavior we illustrate the Bayesian inference results (E, F) that clearly suggest a third order Markov chain model. Finally, this is also confirmed by the cross validation prediction results (G) which again is in accordance with the AIC.

#### Summary

By and large, almost all methods for order selection suggest a Markov chain of order three for the topic sequence in the MSNBC dataset. Again, we can observe that the navigational patterns are not memoryless. Even though this dataset is not a goal-oriented navigation dataset, but is based on free navigation on MSNBC, we can identify similar memory effects as above.

### Structure

In the previous section we observed memory patterns in human navigation over topics in information networks. We are now interested in digging deeper into the structure of human navigational patterns on a topical level. Concretely, we are interested in detecting common navigational sequences and in investigating structural differences between goal-oriented and free form navigation.

First, we want to get a global picture of common transition patterns for each of the datasets. We start with the Markov chain transition matrices, but instead of normalizing the row vectors, we normalize each cell by the complete number of transitions in the dataset. We illustrate these matrices as heatmaps to get insights into the most common transitions in the complete datasets. Due to tractability, we focus on a first order analysis and will focus on higher order patterns later on.

**Figure 8:**
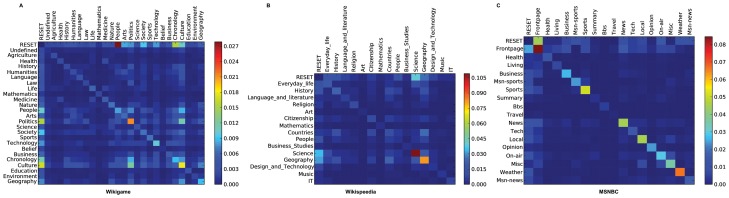
Global structure of human navigation. Common transition patterns of navigational behavior on all three topics datasets (Wikigame, Wikispeedia and MSNBC). Patterns are illustrated by heatmaps calculated on the first order transition matrices. Each cell is normalized by the total number of transitions in the dataset. The vertical lines depict starting states and the horicontal lines depict target states. A main observation is that self transitions – e.g., a transition from *Culture* to *Culture* – are dominating all datasets. However, the goal-oriented datasets (Wikigame and Wikispeedia) exhibit more transitions between distinct categories than the free navigation dataset (MSNBC).

The heatmaps are illustrated in Figure 8. Predominantly, we can observe that self transitions seem to be very common as we can see from the high transition counts in the diagonals of the matrices. This means, that users regularly seem to stay in the same topic while they navigate the Web. Consequently, we might get better representations of the data by using Markov chain models that, instead modeling state transitions in equal time steps, additionally stochastically model the duration times in states (e.g., semi Markov or Markov renewal models). However, we leave these investigations open for future work. For the Wikigame (A) we can observe that the categories *Culture* and *Politics* are the most visited topics throughout the navigational paths. Most of the time the navigational paths start with a page belonging to the *People* topic which is visible by the dark red cell from *RESET* to *People* (remember that the *RESET* state marks both the start and end of a path - see Section “Materials”). However, as this is a game-based goal-oriented navigation scenario, the start node is always predefined. In our second goal-oriented navigation dataset (B) we can see that the paths are dominated by transitions from and to the categories *Science* and *Geography* and there are fewer transitions between other topics. In our MSNBC dataset (C) we can observe that most of the time users remain in the same topic while they navigate and globally no topic changes are dominant. This may be an artifact of the free navigation users practice on MSNBC. Perhaps unsurprisingly, users start with the frontpage most of the time while navigating but do not necessarily come back to it in the end.

**Figure 9:**
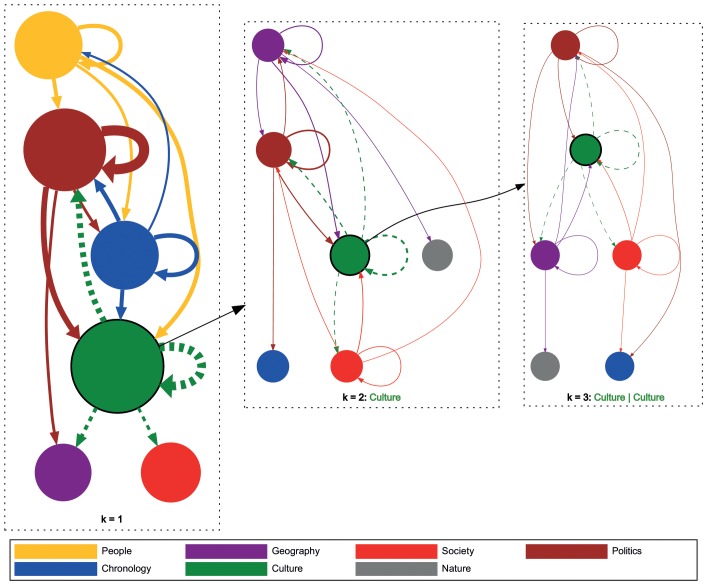
Local structure of navigation for the Wikigame topic dataset. The graphs above illustrate selected state transitions from the Wikigame topic dataset for different 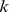 values. The nodes represent categories and the links illustrate transitions between categories. The link weight corresponds to the transition probability from the source to the target node determined by MLE. The node size corresponds to the sum of the incoming transition probabilities from all other nodes to that source node. In the left figure the top four categories with the highest incoming transition probabilities are illustrated for an order of 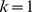. For those nodes we draw the four highest outgoing transition probabilities to other nodes. In the middle figure we visualize the Markov chain of order 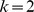 by setting the top topic (*Culture*) as the first click; this diagram shows transition probabilities from top four categories given that users first visited the *Culture* topic. For example, the links from the red node (*Society*) in the bottom-right part of the diagram represent the transition probabilities from the sequence (*Culture*, *Society*). Similarly, we visualize order 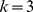 in the right figure by selecting a node with the highest incoming probability (*Culture*, *Culture*) of order 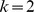. We then show transition probabilities from other nodes given that users already visited (*Culture*, *Culture*). For example, the links from the brown node (*Politics*) at the top represent the transition probabilities from the sequence (*Culture*, *Culture*, *Politics*).

As we have now identified global navigational patterns on the first order transition matrices we turn our attention to models of higher order. Furthermore, we are now interested in investigating local transition probabilities – e.g., being at topic *Science*, what are the transition probabilities to other states. The transition weights directly correspond to the transition probabilities from the source to the target state determined by the MLE (see the section called “Likelihood Method”). We illustrate these local transitional patterns for our Wikigame dataset in Figure 9 (the investigations on the other goal-oriented Wikispeedia dataset exhibit similar patterns, but are omitted due to space limitations). Similar to the observations in Figure 8 we can observe that *Culture* is the most visited topic in our Wikigame dataset. We can now also identify specific prominent topical transition trails. For example, users seem to navigate between *Culture* and *Politics* quite frequently and also vice versa. Contrary, there seem to be specific unidirectional patterns too, e.g., users frequently navigate from *People* to *Politics* but not vice versa. Higher order chains also show similar structure, but on a more detailed level. As previously, the figure also depicts that the vast amount of transitions is between same categories. However, we can now observe that this is also the case for higher order Markov chains – this suggests, that the probability that users stay in the same topic increases with each new click on that topic.

**Figure 10:**
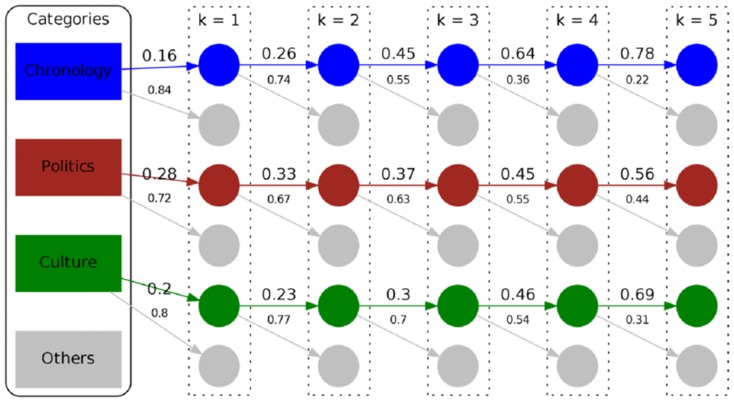
Self transition structure of navigation for the Wikigame topic dataset. The number of times users stay within the same topic vs. the number of times they change the topic during navigation for different orders 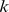 for our Wikigame dataset. Only the top three categories with the highest transition probabilities are shown. With high consistency, the transition probabilities to the same topic increase while those to other categories decrease with ascending order 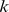.

To further look into this structural pattern, we illustrate the number of times users stay within the same topic vs. the number of times they change the topic during navigation in Figure 10. We can see that the longer the history – i.e., the higher the order of the Markov chain – the more likely people tend to stay in the same topic instead of switching to another topic. We can also see differences regarding this behavior between distinct categories; e.g., users are more likely to stay in the topic *Chronology* than in the topic *Politics* the higher the order is. For our Wikispeedia dataset we can observe similar patterns – i.e., the higher the order the higher the chance to stay in the same topic.

**Figure 11:**
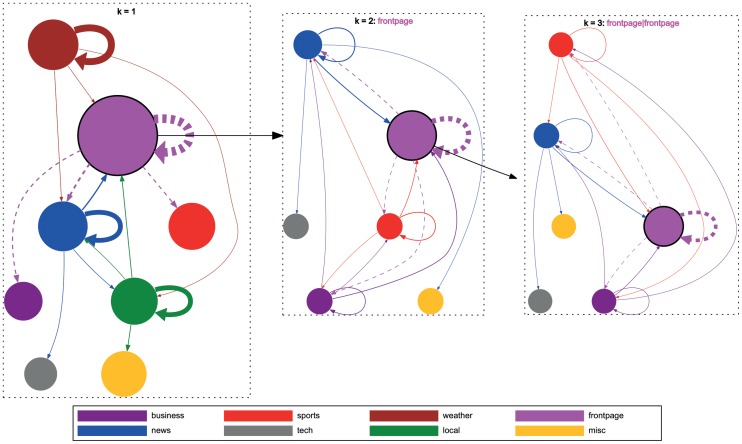
Local structure of navigation for the MSNBC dataset. The graphs above illustrate selected state transitions from the MSNBC dataset for different 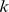 values. The nodes represent categories and the links illustrate transitions between categories. The link weight corresponds to the transition probability from the source to the target node determined by MLE. The node size represents the global importance of a node in the whole dataset and corresponds to the sum of the outgoing transition probabilities from that node to all other nodes. For visualization reasons we primarily focus on the top four categories with the highest sum of outgoing transition probabilities – i.e., those with the largest node sizes – for an order of 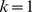. For those nodes we draw the four highest outgoing transition probabilities to other nodes. In the middle figure we visualize the Markov chain of order 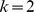 by setting the top topic (frontpage) from order 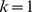 as the first click; this diagram shows transition probabilities from top four categories given that users first visited the frontpage topic (represented by the dashed transitions in the left figure representing 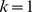). For example, the links from the blue node (news) in the top-left corner of the diagram represent the transition probabilities from the sequence (frontpage, news) to other nodes. Similarly, we visualize order 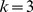 in the right figure by selecting a node with the highest sum of outgoing transition probabilities (frontpage, frontpage) and its four highest outgoing transition probabilities from order 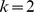 (represented by the dashed transitions in the middle figure representing 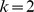). We then show transition probabilities from other nodes given that users already visited (frontpage, frontpage). For example, the links from the red node (sports) at the top represent the transition probabilities from the sequence (frontpage, frontpage, sports) to other nodes.

**Figure 12:**
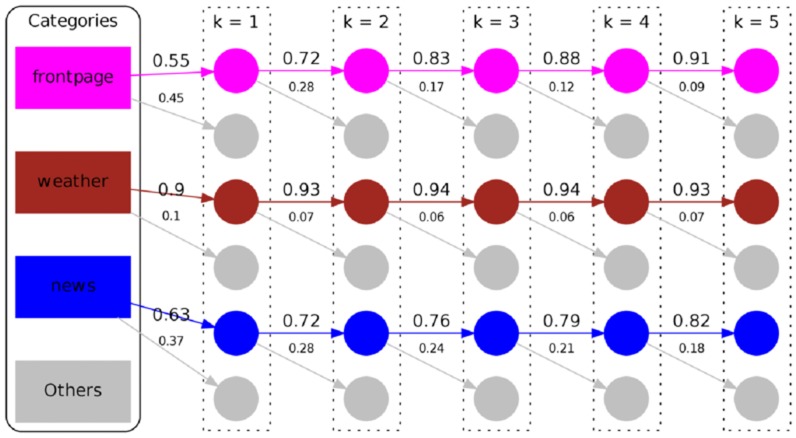
Self transition structure of navigation for the MSNBC dataset. The number of times users stay within the same topic vs. the number of times they change the topic during navigation for different values of 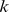. Only the top three categories with the highest transition probabilities are shown. With high consistency, the transition probabilities to the same topic increase while those to other categories decrease with ascending order 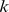.

In order to contrast goal-oriented and free-form navigation, we also depict state transitions in similar fashion derived from the MSNBC dataset in Figure 11. In this figure we can see that the topic *business* is the most used. To give a navigational example: users frequently navigate from *business* to *news* and vice versa. However, there are also navigational patterns just going one direction. For example, users seem to frequently navigate from *business* to *sports* but not in the opposite direction. Again, higher order chains show similar patterns. Like in the Wikigame topic dataset we can as well observe that most of the transitions seem to be between similar categories. In Figure 12 we depict the number of times a user stays in the same topic vs. the number of times she switches the topic for the categories with the highest transition probabilities. We can again observe that the higher the Markov chain the more likely people tend to stay in the same topic while navigating. Nevertheless, an interesting difference to the Wikigame topic dataset can be observed. Concretely, we can see that the probability of staying in the same topic is much higher for the MSNBC dataset. Especially, the topic *weather* exhibits a very high probability of staying in the same topic (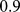 for 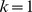). A possible explanation is that users navigate on a semantically more narrow path on MSNBC. If you are interested about the weather you just check the specific pages on MSNBC while on Wikipedia you might get distracted by different categories at a higher probability. So these concrete observations seem to be very specific for the Web site and domains of the site users navigate on while the general patterns seem to be applicable for both of our datasets at hand.

## Discussion

Our findings and observations in this article show that simple likelihood investigations (see e.g., [12]) may not be sufficient to select the appropriate order of Markov chains and to prove or falsify whether human navigation is memoryless or not. To ultimately answer this, we think it is inevitable to look deeper into the results obtained and to investigate them with a broader spectrum of model selection methods starting with the ones presented in this work.

By applying these methods to human navigational data, the results suggest that on the Wikigame page dataset a zero order model should be preferred. This is due to the rising complexity of higher order models and indicates that it is difficult to derive the appropriate order for finite datasets with a huge amount of distinct pages having only limited observations of human navigational behavior. In this article we presented and applied a variety of distinct model selection that all include (necessary) ways of penalizing the large number of parameters needed for higher order models. Yet, we do not necessarily know what would happen if we would apply the models to a much larger number of navigational paths over pages. Perhaps higher order models would then outperform lower ones. As it is unlikely to get hands on such an amount of data for large websites, a starting point to further test this could be to analyze a sub-domain with rich data; i.e., a large number of observations over just a very limited number of distinct pages. However, due to no current access to such data, we leave this open for future work.

On the other hand, the results on a topical level are intriguing and show a much clearer picture: They suggest that the navigational patterns are not memoryless. Higher order Markov chains – i.e., second or third order – seem to be the most appropriate. Henceforth, the navigation history of users seem to span at least two or three states on a topical level. This gives high indications that common strategies (at least on a topical level) exist among users navigating information networks on the Web. It is certainly intriguing to see similar memory patterns in both goal-oriented navigation (Wikigame and Wikispeedia) and free form navigation (MSNBC), and different kinds of systems (encylopedia vs. news portal).

In order to confirm that these observed memory effects are based on the actual human navigation patterns we again look at our random path dataset introduced in the section entitled “Likelihood Method” with the log-likelihoods visualized in Figure 2. We can recapitalize, that these simple log-likelihoods would suggest a higher order model for the randomly produced navigational patterns. However, if we apply our various model selection techniques the results suggest a zero or at maximum a first order Markov chain model which is the logic conclusion for this random process. Hence, this confirms that our observations on the real nature navigational data are based on human navigational memory patterns and would not be present in a random process.

**Figure 13:**
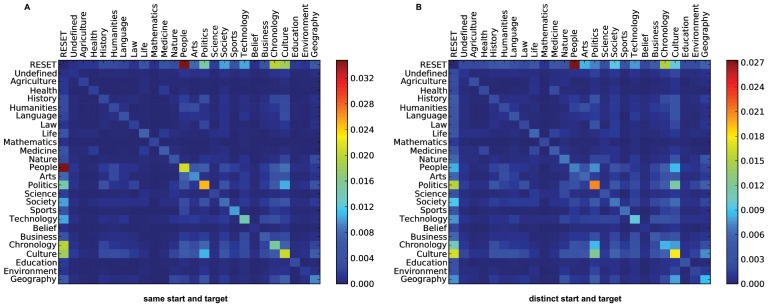
Common global transition patterns of navigational behavior on the Wikigame topic dataset. The results should be compare with Figure 8. The results are split by only looking at a corpus of paths where each path starts with the same topic as it ends (A) and by looking at a corpus with distinct start and target categories (B).

Finally, we showed in the section called “Structure” that common structure in the navigational trails exist among many users – i.e., common sequences of navigational transitions. First of all, we could observe that transitions between the same topic are common among all three datasets. However, they occur more frequently in our free form navigational data (MSNBC) than in the goal-oriented navigation datasets (Wikigame and Wikispeedia). Furthermore, users also seem to be more likely to stay longer in the same topic while navigating MSNBC while they seem to switch categories more frequently in both the Wikigame and Wikispeedia datasets. A possible explanation for this user behavior might be that users on MSNBC are more driven by specific information needs regarding one topic. For example, a user might visit the website to get information about the weather only. Contrary, exact information goals on Wikipedia might not always be in the same topic. Suppose, you are located on *Seoul* which belongs to the *Geography* topic and you want to know more about important inventions made in *Seoul*. A possible path then could be that you navigate over a *People* topic page and finally reach a *Science* topic page. However, we need to keep in mind that our goal-oriented datasets are based on game data with predefined start and target nodes. This means, that if the target nodes regularly lie in distinct categories, the user might be forced to switch categories more frequently. To rule this out, we illustrate the heatmap of our Wikigame dataset (cf. Figure 8) again by splitting the path corpus into two parts (see Figure 13): (A) only considering paths where the start and target node lie in the same topic and (B) only taking paths with distinct start and target categories. If the bias of given start and target nodes would influence our observations for specific structural properties of goal-oriented navigational patterns, Figure 13 would show strong dissimilarities between both illustrations which is not the case. Hence, we can state with strong confidence that the differences between goal-oriented and free form navigation stated in this section are truly based on the distinct strategies and navigational scenarios. Nevertheless, we also need to keep in mind that the website design and inherent link structure (Wikipedia vs. MSNBC) might also influence this behavior. For example, a reason could be that Wikipedia has more direct links between distinct categories in comparison to MSNBC or that Wikipedia's historical coverage steers user behavior to specific kinds of navigational patterns. To explicitly rule this possibility out, we would need to investigate the underlying link networks in greater detail, which we leave open for future work. We also plan on looking at data capturing navigational paths over distinct platforms of the Web (e.g., from toolbar data) which may allow us to make even more generic statements about human navigation on the Web.

## Conclusions

This work presented an extensive view on detecting memory and structure in human navigational patterns. We leveraged Markov chain models of varying order for detecting memory of human navigation and took a thorough look at structural properties of human navigation by investigating Markov chain transition matrices.

We developed an open source framework (https://github.com/psinger/PathTools) [20] for detecting memory of human navigational patterns by calculating the appropriate Markov chain order using four different, yet complementary, approaches (likelihood, Bayesian, information-theoretic and cross validation methods). In this article we thoroughly present each method and emphasize strengths, weaknesses and relations between them. By applying this framework to actual human navigational data we find that it is indeed difficult to make plausible statements about the appropriate order of a Markov chain having insufficient data but a vast amount of states which results in too complex models. However, by representing pages by their corresponding topic we could identify that navigation on a topical level is not memoryless – an order of two and respectively three best explain the observed data, independent whether the navigation is goal-oriented or free-form. Finally, our structural investigations illustrated that users tend to stay in the same topic while navigating. However, this is much more frequent for our free form navigational dataset (MSNBC) as compared to both of the goal-oriented datasets (Wikigame and Wikispeedia).

Future attempts of modeling human behavior in the Web can benefit from the methodological framework presented in this work to thoroughly investigate such behavior. If one wants to resort to a single model selection technique, we would recommend to use the Bayesian approach if computationally feasible.

Our work strongly indicates memory effects of human navigational patterns on a topical level. Such observations as well as detailed insights into structural regularities in human navigation patterns can e.g., be useful for improving recommendation systems, web site design as well as faceted browsing. In future work, we want to extend our ideas of representing Web pages with categories by looking at further features for representation. We also plan on tapping into the usefulness of further Markov models like the hidden Markov model, varying order Markov model or semi Markov model. Also, we want to improve recommendation algorithms by the insights generated in this work and explore the implications higher order Markov chain models may have on ranking algorithms like PageRank.
