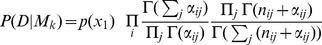# Correction: Detecting Memory and Structure in Human Navigation Patterns Using Markov Chain Models of Varying Order

**DOI:** 10.1371/journal.pone.0114952

**Published:** 2014-12-01

**Authors:** The PLOS ONE Staff

The 20^th^ equation in the Evidence section of the Methods is incorrect. This error was introduced during the preparation of this manuscript for publication. The publisher apologizes for this error. Please see the complete, correct equation here: